# Functional redundancy and niche complementarity maintain nitrification stability in rapid sand filters

**DOI:** 10.3389/fmicb.2025.1741059

**Published:** 2026-01-21

**Authors:** Alejandro Palomo, S. Jane Fowler, Ibrahim M. Nemer, Borja Valverde-Pérez, Yan Zheng, Yunjie Ma, Barth F. Smets

**Affiliations:** 1 State Key Laboratory of Soil Pollution Control and Safety, Southern University of Science and Technology, Shenzhen, China; 2 Guangdong Provincial Key Laboratory of Soil and Groundwater Pollution Control, School of Environmental Science and Engineering, Southern University of Science and Technology, Shenzhen, China; 3 Department of Environmental and Resource Engineering, Technical University of Denmark, Kgs. Lyngby, Denmark; 4 Department of Biological Sciences, Simon Fraser University, Burnaby, BC, Canada; 5 Department of Biological and Chemical Engineering, Aarhus University, Aarhus, Denmark

**Keywords:** ammonia oxidation archaea (AOA), ammonia oxidation bacteria (AOB), comammox, functional redundancy, niche complementarity, nitrification, nitrospira, rapid sand filter (RSF)

## Abstract

Nitrification in drinking water biofilters is mediated by a complex guild of ammonia oxidizers, yet the mechanisms facilitating the coexistence of these functionally redundant nitrifier guild members are not well understood. Using lab-scale columns packed with material from a full-scale groundwater-fed rapid sand filter (RSF), we investigated the responses of co-occurring complete ammonia-oxidizing (comammox) *Nitrospira*, ammonia-oxidizing bacteria (AOB), and archaea (AOA) to operational disturbances over 30 days. Overall, robust nitrification was maintained, with ammonium removal scaling proportionally with substrate loading under oxic conditions. A marked depth-dependent differentiation of nitrifier biomass was identified in the source filter; a 66-fold enrichment of *Nitrospira* in the top layer determined initial removal capacity, while the bottom layer adapted through both nitrifier proliferation and load-dependent physiological upregulation. Bacterial nitrifiers were primarily structured by strong legacy effects and spatial gradients. AOA abundance, however, was independent of layer origin and governed by environmental conditions, exhibiting a preference for colder (10 °C) and oxygen-limited conditions. Substrate-dependent niche partitioning based on free-ammonia concentration was evident among all nitrifier guild members, enabling stable coexistence without competitive exclusion. Further, temperature-dependent turnover between comammox clades A and B occurred independently of substrate or oxygen conditions, suggesting fine-scale ecological partitioning between phylogenetically distinct comammox species. These findings indicate that functional redundancy, maintained by niche complementarity among diverse members of the nitrifier guild, underpins the stability of RSF nitrification under environmental disturbances. This study provides a mechanistic framework for understanding microbial coexistence in engineered oligotrophic ecosystems facing fluctuating environmental conditions.

## Introduction

Nitrification, the biological conversion of ammonium (NH_4_^+^) to nitrate (NO_3_^−^), is a crucial process in the nitrogen cycle with significant implications for ecosystems ranging from soils and oceans to engineered water treatment systems. For over a century, this process was understood as a strict division of labor between two distinct microbial guilds: ammonia-oxidizing bacteria (AOB) and archaea (AOA) that perform the first step to nitrite (NO_2_^−^), and nitrite-oxidizing bacteria (NOB) that complete the oxidation to nitrate. The discovery of complete ammonia oxidizers (comammox) within the genus *Nitrospira* (previously known only as canonical nitrite oxidizers) profoundly altered this view by demonstrating that single organisms can catalyze the full transformation from NH₃ to NO₃^−^ (Daims et al., 2015; van Kessel et al., 2015). Since their discovery, comammox *Nitrospira* have been detected in a wide array of environments, including soils, sediments, wastewater and drinking-water biofilters (Pinto et al., 2015; Orellana et al., 2017; Sun et al., 2020; Palomo et al., 2022b; Zheng et al., 2023) and often constitute a numerically significant portion of the ammonia-oxidizing guild, particularly in biofilms or oligotrophic systems (Fowler et al., 2018). Comammox are phylogenetically divided into two major clades (clade A and clade B) based on their ammonia monooxygenase phylogeny (Palomo et al., 2018), yet both clades are often detected simultaneously across a wide range of environments (Pjevac et al., 2017; Xia et al., 2018).

The coexistence and uneven distribution of these nitrifiers in ecosystems indicates niche differentiation among AOB, AOA and comammox *Nitrospira* (Palomo et al., 2022a; Chai et al., 2025). Specifically, niche separation between AOB and AOA is often attributed to environmental factors such as ammonium concentration and pH, with AOA being better adapted to low-substrate and acidic environments (Verhamme et al., 2011; Zhang et al., 2012).

Similarly, Costa et al. (2006) proposed that substrate-limited conditions would favor comammox over AOB due to potentially higher biomass growth yield (Costa et al., 2006). Additionally, experimental data revealed that comammox organisms possessed low Km values (Kits et al., 2017; Sakoula et al., 2021). Consistent with these theoretical predictions and experimental observations, subsequent research has shown comammox dominance in oligotrophic or substrate-limited systems (Gülay et al., 2019; McKnight and Neufeld, 2024) and biofilms under oxygen limitation (Xiang et al., 2025), while AOB dominated in ammonia-richer environments (Yang et al., 2022; Vilardi et al., 2024). However, further experiments are necessary to validate these patterns and explore other fluctuating environmental factors, such as temperature and water chemistry (e.g., pH, alkalinity, organic matter content), which may differentiate the abundances and activities of the diverse members of the nitrifier guild (Zhou et al., 2021).

While both comammox *Nitrospira* clades A and B perform complete ammonia oxidation, genomic and ecological evidence indicates that they have evolved distinct life strategies (Palomo et al., 2022b). Clade A is commonly detected in environments with higher ammonia availability, such as wastewater treatment plants (Cotto et al., 2020; Spasov et al., 2020), whereas clade B is frequently dominant in oligotrophic systems like groundwater-fed biofilters (Fowler et al., 2018). In addition, clade B harbors MEP-type ammonia transporters, also found in AOA, which are characterized by high ammonia affinity (micromolar-range). In contrast, clade A contains Rh-type transporters similar to those in AOB, which have lower ammonia affinity but higher capacity (Palomo et al., 2018). Despite these genomic predictions, experimental validation awaits the cultivation of clade B representatives, as all comammox strains isolated to date belong to clade A (Kits et al., 2017; Sakoula et al., 2021).

Genomic analyses have also suggested metabolic versatility beyond ammonia oxidation in comammox *Nitrospira*. Numerous comammox genomes encode pathways for uptake and catabolism of simple organic compounds (Kop et al., 2025), suggesting potential for mixotrophic metabolism. Experimental studies have documented the utilization of various organic substrates by different *Nitrospira* species (Daims et al., 2001; Koch et al., 2015; Lawson et al., 2021), though the ecological significance and extent of mixotrophy in environmental comammox populations remain uncertain.

Rapid sand filters (RSFs) in drinking water treatment represent ideal model systems for studying microbial interactions, as they contain stratified communities under low-nutrient, disturbance-prone conditions (Tatari et al., 2016; Hu et al., 2020), and are also known to harbor a diversity of comammox *Nitrospira* clades A and B, AOB, and AOA (Fowler et al., 2018; Palomo et al., 2022a). In this study, we conducted lab-scale experiments using materials from a full-scale RSF that treats groundwater with a diverse nitrifying guild (Palomo et al., 2016). The objective was to investigate competition and differential abundance of members within nitrifying guilds under short-term disturbances of different ammonium loadings, oxygen availability, temperature, and the addition of an external carbon source. We hypothesized that (1) increased ammonium loading would favor canonical AOB and/or comammox clade A taxa, whereas low loading would favor clade B and AOA; (2) oxygen limitation would selectively benefit comammox; and (3) acetate addition would select for comammox organisms with mixotrophic capacity. To test these hypotheses, we combined qPCR targeted to nitrifier groups, high-resolution 16S rRNA gene amplicon sequencing, and differential abundance analyses.

## Materials and methods

### Sample collection and site description

Filter material and water samples were collected from Islevbro waterworks (Copenhagen, Denmark), a groundwater-fed rapid sand filter system described previously (Lee et al., 2014). Briefly, raw water abstracted from a deep chalk aquifer undergoes aeration, iron oxidation in a retention tank (~20 min contact time), and dual-stage filtration (pre-filter for Fe-hydroxide retention; after-filter for biological ammonium removal). After-filters (~0.7 m depth, 1 mm grain diameter, ~30 years operation, backwashed every 14 days) receive influent with ~0.13 mg NH₄^+^-N/L, 9.3 mg/L dissolved oxygen (DO), pH 7.3, and 9–11 °C temperature, achieving ammonium removal to <0.01 mg/L. Filter material from one after-filter was core-sampled midway between backwash events using a 60 cm plexiglass cylinder, extruded, and aseptically sliced into depth sections: 0–10 cm (top), 10–40 cm (middle), and 40–50 cm (bottom). Top and bottom materials were used for column experiments and stored wet at 4 °C during transportation. Subsamples for DNA extraction were stored at −80 °C.

### Lab-scale column setup and experimental design

A lab-scale column assay, detailed elsewhere (Tatari et al., 2013), was used to investigate ammonium removal under varied conditions. Briefly, plexiglass columns (5 cm bed height, 2.6 cm inner diameter) were packed with sand and continuously fed with water. Two independent experiments, each comprising eight parallel columns, were operated for 30 days (Table 1 and Supplementary Figure S1). Column systems were placed in a temperature-controlled room set at either 10 °C (Experiment 2) or kept at room temperature (mean ≈ 20 °C; Experiment 1). For Experiment 1 (20 °C), columns were packed with either top- or bottom-layer material and fed with after-filter effluent water spiked with ammonium (as NH₄Cl) at a reference loading rate of 35 g NH₄^+^-N/m^3^ filter material/d (1.46 g NH₄^+^-N/m^3^/h; 1 mg/L NH₄^+^-N at 0.96 L/d flowrate), equivalent to full-scale conditions. Additional loading rates included 0.1 × reference (0.1 mg/L NH₄^+^-N) and 5 × reference, achieved either by increasing influent NH₄^+^-N concentration to 5 mg/L (oxygen-limited condition) or by increasing flowrate fivefold (non-limiting oxygen condition). The high-concentration treatment was designated oxygen-limited because complete nitrification of 5 mg/L NH₄^+^-N requires 22.9 mg O₂/L (stoichiometric demand of 4.57 mg O₂ per mg NH₄^+^-N oxidized), exceeding the measured influent dissolved oxygen of 10.6 ± 0.8 mg/L. For Experiment 2 (10 °C), columns packed with top-layer material were fed with three different waters to evaluate organic carbon effects on nitrifier-heterotroph interactions: after-filter effluent spiked with NH₄Cl (minimal organic carbon control), after-filter effluent spiked with NH₄Cl + 1.5 mg/L sodium acetate (labile carbon amendment), or pre-filter effluent spiked with NH₄Cl (containing natural groundwater dissolved organic matter (DOM)). In this experiment, 5× loading rates were set by increasing NH₄^+^-N concentration to 5 mg/L (oxygen-limited condition as described in Experiment 1). Four times during each experiment, 2 g of filter material was sampled from each column for microbial characterization (qPCR, 16S rRNA gene amplicon sequencing), increasing the volumetric loading rate by 52 ± 13% over the experimental period due to material removal.

**Table 1:** Summary of experimental design.

Column no.	Column ID	Temp (°C)	Source material	NH₄^+^ loading rate^a^	O₂ limitation	Water source
Experiment 1						
1	CB20_1	20	Bottom	0.1×	No	After-filter effluent
2	CB20_2			1×	No	
3	CB20_3			5×	No	
4	CB20_4			5×	Yes	
5	CT20_1		Top	0.1×	No	
6	CT20_2			1×	No	
7	CT20_3			5×	No	
8	CT20_4			5×	Yes	
Experiment 2						
1	CT10_1	10	Top	1×	No	After-filter effluent
2	CT10_2a					After-filter influent
3	CT10_2b					After-filter influent
4	CT10_3a					After-filter effluent + Acetate
5	CT10_3b					After-filter effluent + Acetate
6	CT10_4a			5×	Yes	After-filter influent
7	CT10_4b					After-filter influent
8	CT10_5					After-filter effluent + Acetate

^a^NH_4_^+^ loading rate: 1 × indicates the reference volumetric loading rate of 35 g NH₄^+^-N/m3 filter material/d equivalent to the full-scale conditions, (5×, O₂ Limitation_No) condition was the loading increased 5 × by raising the flow rate, and (5×, O₂ Limitation_Yes) was the loading increased 5 × by raising influent NH₄^+^ concentration.

### Physiochemical analysis

Duplicate water samples were collected along the experiments every 2–4 days, filtered (Sartorius Minisart 0.20 μm), and stored at −20 °C. In Experiment 1, NH₄^+^, NO₂^−^, and NO₃^−^ were analyzed using an autoanalyzer (Bran+Luebbe Analytics, 2012). In Experiment 2, NH₄^+^ was measured using a salicylate-hypochlorite method (Bower and Holm-Hansen, 1980), NO₂^−^ via an adapted Grasshoff et al. (1983) method, and NO₃^−^ using a Merck Spectroquant test kit 109,713. Different methods were used due to equipment availability, with cross-calibration ensuring consistency. DO (influent and effluent) was periodically measured with a handheld meter (WTW, Multi 3,430, with FDO® 925) during Experiment 2.

### DNA extraction, quantification, and sequencing

Filter material from the top of the columns was sampled four times during the 30-day period, with the final sample at day 30. Samples were collected in cryotubes, flash-frozen in liquid nitrogen, and stored at −80 °C. DNA was extracted from the 0.5 g sand collected just before and at the end of the experiments using the FastDNA® Spin Kit for Soil (MP Biomedicals, Santa Ana, CA, USA) per the manufacturer’s instructions. DNA concentration and purity were assessed using a NanoDropTM 2000 Spectrophotometer (Thermo Scientific, Wilmington, DE, USA). Quantitative PCR (qPCR) assays were performed in duplicate using a Chromo 4 Thermal Cycler (Bio-Rad Laboratories, Hercules, CA, USA). Each reaction contained 12.5 μL 2 × iQ SYBR Green Supermix, 20 μM forward and reverse primers, 10 ng DNA template, and PCR-grade water. Targeted groups included total bacteria (16S rRNA gene), *Nitrospira* (specific 16S rRNA region), AOB (specific 16S rRNA region), and AOA (*amoA* gene) (Supplementary Table S1). Cell density calculations assumed one copy per cell, except for the 16S rRNA of the total community, where copy numbers were estimated using CaRcone (R script^1^). DNA was PCR-amplified using primers PRK341F (5′-CCTAYGGGRBG CASCAG-3′) and PRK806R (5′-GGACTACNNGGGTATCTAAT-3′) for 35 cycles to target the V3-V4 hypervariable region (Yu et al., 2005). PCR products were purified and sequenced on the Illumina MiSeq platform at the DTU MultiAssay Core Centre (Lyngby, Denmark).

### Estimation of cell-specific ammonium oxidation rates

To evaluate physiological adaptation versus biomass growth, apparent cell-specific ammonium oxidation rates (SAOR; fmol NH₄^+^-N/cell/h) were estimated for the initial and final experimental phases. SAOR was calculated by dividing the volumetric ammonium removal rate (g NH₄^+^-N/m^3^/h) by the total nitrifier cell density (sum of AOB, AOA, and *Nitrospira*) converted to a volumetric basis using layer-specific filter material bulk densities of 1.1 g/cm^3^ for the top layer and 1.7 g/cm^3^ for the bottom layer, as previously reported for this system (Lee et al., 2014). Initial SAOR was calculated using average removal rates from the first 48 h and the nitrifier density of the corresponding inoculum (top or bottom material). Final SAOR used average removal rates from days 28–30 and the column-specific cell densities measured at day 30.

### Sequence analysis

16S rRNA gene amplicon libraries were processed using the DADA2 pipeline (Callahan et al., 2016), which outputs the abundance of error-corrected amplicon sequence variants (ASVs). ASVs were classified with the SILVA prokaryotic reference database v132. After filtering, 1.2 million sequences were retained, averaging 65,000 sequences per sample. Further analysis was carried out in R packages phyloseq (McMurdie and Holmes, 2013) and ampvis2 (Andersen et al., 2018). Statistically significant differences in sequences before and after 30-day incubations were identified using DESeq2 (Wald significance test, parametric fit type, padj < 0.05) (Love et al., 2014).

### Assignment of *Nitrospira* ASVs to Comammox and canonical lineages

The 16S rRNA gene generally provides limited phylogenetic resolution for distinguishing comammox *Nitrospira* from canonical nitrite oxidizers (Daims et al., 2015; Lawson and Lücker, 2018). However, extensive prior characterization of the filter used for our column experiment, including metagenomic sequencing, genome binning, and recovery of *Nitrospira* metagenome-assembled genomes (MAGs) (Palomo et al., 2016, 2018, 2022a) and *amoA*-targeted qPCR and sequencing (Fowler et al., 2018), enabled us to develop a robust classification framework for *Nitrospira* ASVs based on phylogenetic placement and cross-validation with functional gene data. MAGs previously recovered from this system had been functionally annotated for comammox-specific genes, including ammonia monooxygenase (*amoA*) and hydroxylamine dehydrogenase (*hao*), enabling reliable distinction between comammox and canonical NOB, as well as between comammox clades A and B. For each MAG, we extracted the corresponding 16S rRNA gene sequence (when available) and aligned these sequences using MUSCLE v3.8.31 (Edgar, 2004) together with *Nitrospira*-affiliated ASVs from the present study and 16S rRNA gene sequences retrieved from publicly available *Nitrospira* genomes. These alignments were used to construct a maximum-likelihood phylogenetic tree using RAxML v8.2.11 (Stamatakis, 2014) with 550 rapid bootstraps (determined using the autoMRE option) and the GTRCAT substitution model (best model determined using jModelTest v.2.1.10; Posada, 2008). The tree was rooted using *Leptospirillum* species as outgroup and visualized using the Interactive Tree of Life (iTOL) web tool (Letunic and Bork, 2016). Each ASV was assigned to a category (comammox clade A, comammox clade B, or canonical NOB) based on its closest phylogenetic placement relative to MAG-derived 16S rRNA genes with known metabolic identity. Assignments were validated by BLASTn searches against the NCBI Whole Genome Shotgun contigs (WGS) database, confirming that top hits aligned with tree-based classifications (Supplementary Table S2). To further validate our classifications, we compared the relative abundances of comammox versus canonical *Nitrospira*, and comammox clade A versus clade B proportions, derived from 16S rRNA gene amplicon sequencing in this study with independent estimates from previous investigations of the same filter using: (1) metagenomic read recruitment, (2) qPCR targeting *Nitrospira* 16S rRNA genes and comammox-specific *amoA* genes to determine the proportion of comammox within total *Nitrospira*, and (3) comammox *amoA* amplicon sequencing to distinguish clade A versus clade B ratios. Amplicon-derived, qPCR-derived, and metagenome-derived abundance estimates showed strong concordance (Supplementary Table S3). Furthermore, direct comparison of individual MAG and ASV abundances revealed strong agreement (R^2^ = 0.93, Supplementary Figure S2), including consistent rank ordering of dominant clade B, intermediate clade A, and low-abundance canonical NOB lineages. This multi-method validation supports the reliability of our 16S rRNA gene-based *Nitrospira* classifications in this well-characterized system.

### Statistical analysis

Statistical analyses were performed in R version 4.5.1. Volumetric removal rates (g NH₄^+^-N/m^3^/h) were analyzed using linear mixed-effects models (lme4 package; Bates et al., 2015) with column identity as a random intercept to account for repeated measurements. A primary model included loading rate, temperature, layer origin, oxygen availability, and water source as fixed effects: Removal Rate ~ Loading_Rate + Temperature + Layer + Oxygen + Water_Source + (1|Column). Because the experimental design was partially confounded, focused subset analyses were conducted to obtain unbiased factor estimates, using Welch’s t-tests for pairwise contrasts. Fixed-effect significance was assessed by Type III ANOVA with Satterthwaite’s approximation (package lmerTest; Kuznetsova et al., 2017). *Post hoc* comparisons were performed using estimated marginal means with Tukey’s HSD adjustment (package emmeans; Lenth and Piaskowski, 2017). Model performance was evaluated based on marginal and conditional R^2^ values. To analyze differences in microbial cell densities, data were log₁₀-transformed and analyzed using separate linear models for each microbial group. These models included temperature, layer origin, oxygen availability, loading rate, and water source as fixed effects. The significance of factors was assessed using a Type III ANOVA, with significant differences explored through pairwise comparisons of estimated marginal means adjusted with Tukey’s HSD. For all models, assumptions of normality (Shapiro–Wilk test) and homoscedasticity (Breusch-Pagan test) were met (all *p* > 0.17). Fold-changes in cell density relative to the source material (FC = Final/Source) were calculated, log₂-transformed, and analyzed using linear models. For all statistical tests, significance was set at *α* = 0.05.

## Results

### Ammonium removal performance under variable operating conditions

**Figure 1:**
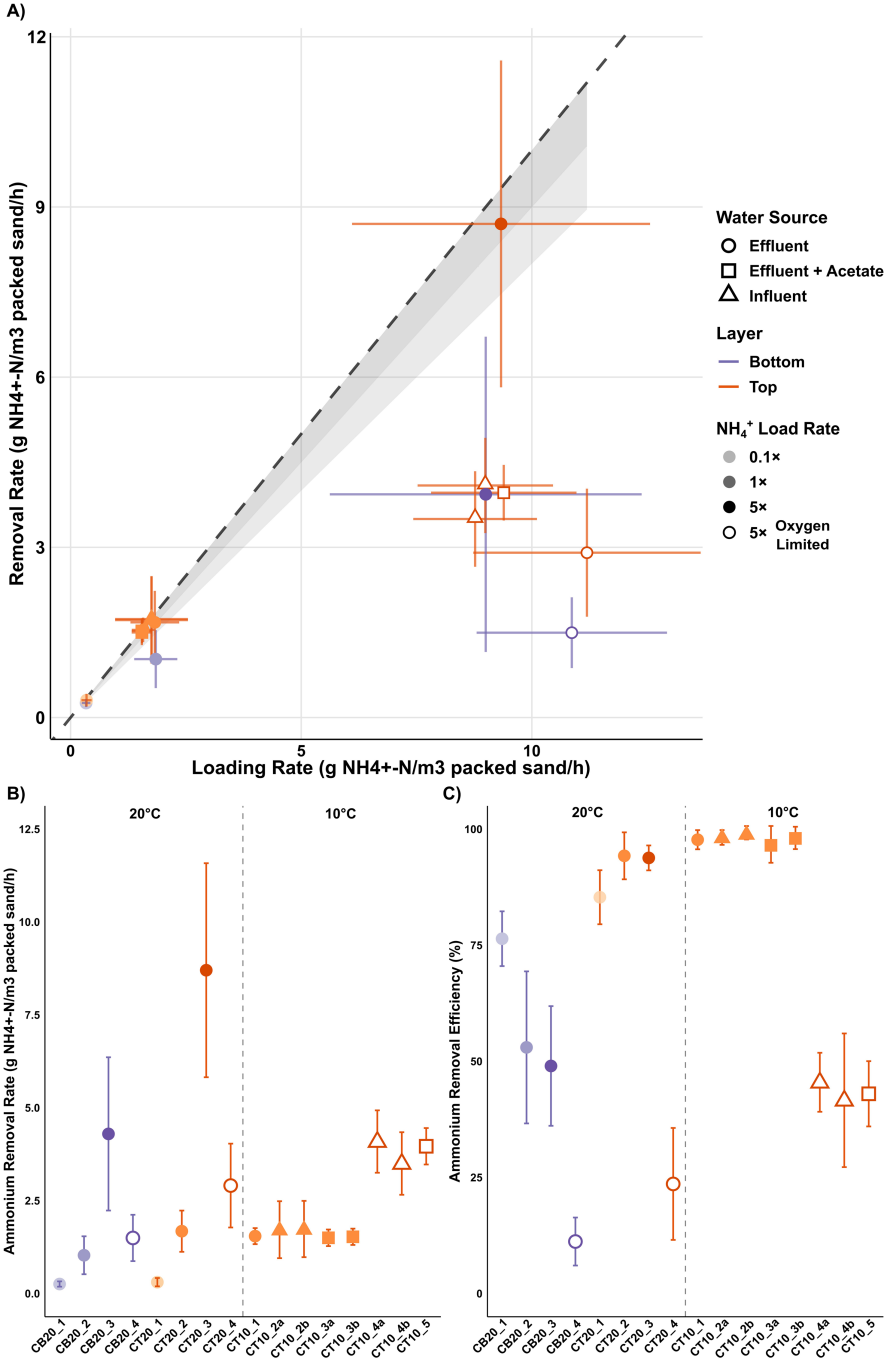
**(A)** Relationship between ammonium loading and removal rate, with shaded bands indicating 80–90% (light gray) and 90–100% (dark gray) removal efficiency relative to the complete removal 1:1 line (dashed). **(B)** Ammonium removal rate during the 30 day experiment. **(C)** Ammonium removal efficiency during the 30 day experiment. Detailed operational conditions for each column are described in Table 1.

Rapid sand filters have been operated for groundwater treatment at Islevbro waterworks since 1923, achieving complete ammonium removal from influent concentrations of ~0.13 ± 0.05 mg NH₄^+^-N/L to < 0.01 mg NH₄^+^-N/L in effluents. Lab-scale columns packed with top-layer (0–10 cm depth) or bottom-layer (40–50 cm depth) filter material from Islevbro were operated under varying conditions to investigate RSF microbial community responses to short-term disturbances, including temperature (10 °C vs. 20 °C), oxygen availability (adequate vs. limiting), water source (after-filter effluent, acetate-amended after-filter effluent, or after-filter influent), and ammonium loading rates (0.1×, 1×, 5 × reference loading of 1.46 g NH₄^+^-N/m^3^/h, equivalent to full-scale conditions) (Table 1). Results showed that ammonium loading emerged as the primary determinant of volumetric removal capacity (*p* < 0.001), with rates scaling proportionally from 0.3 ± 0.1 g NH₄^+^-N/m^3^/h at 0.1 × loading to 7.1 ± 3.3 g NH₄^+^-N/m^3^/h at 5 × loading under non-oxygen-limiting conditions (Figure 1A). Columns packed with top-layer material achieved peak rates of 8.7 ± 2.9 g NH₄^+^-N/m^3^/h with 94 ± 3% removal efficiency under high-flow, 5 × loading conditions (Figure 1B). Under identical ammonium loading but oxygen-limiting operation, removal rates dropped to 3.7 ± 0.9 g NH₄^+^-N/m^3^/h (*p* < 0.05; Figure 1B). This oxygen limitation was stoichiometric rather than kinetic: complete oxidation of 5 mg NH₄^+^-N/L requires 22.9 mg O₂/L, whereas influent contained only 10.7 ± 0.2 mg/L of oxygen. Effluent oxygen concentrations confirmed near-complete oxygen depletion in O₂-limited columns (1.1 ± 0.7 mg/L) compared with oxic controls (4.8 ± 0.3 mg/L), suggesting operation at maximal O₂-limited capacity.

Filter layer origin also significantly affected nitrification performance (p < 0.05), with top-layer columns consistently outperforming those packed with bottom-layer material, particularly at the high loading (Figure 1). Notably, bottom-layer removal rates and efficiencies increased after the first few days (Supplementary Figure S3). By contrast, temperature and water source had no significant effects on ammonium removal (*p* > 0.6). Across all treatments, nitrification proceeded to completion with negligible nitrite accumulation, except during a transient lag phase in the first 24–48 h of high loading operation (Supplementary Figure S4).

### Source material exhibits strong depth-dependent differences in nitrifier populations

**Figure 2:**
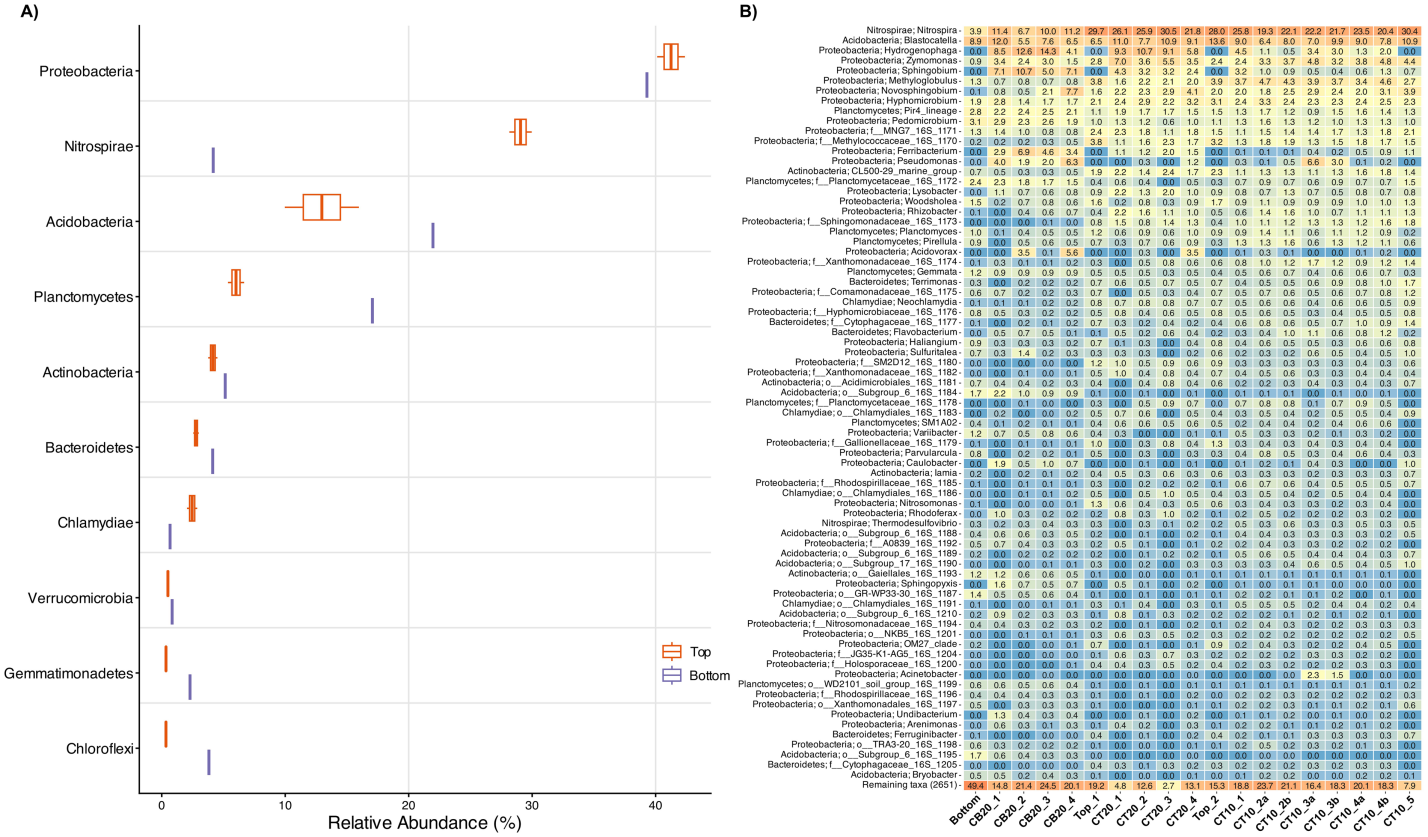
**(A)** Relative abundance of the top 10 most abundant phyla for the initial top (*n* = 2; orange color) and bottom (*n* = 1; purple color) material based on 16S rRNA gene amplicon sequencing. **(B)** Relative abundance of the top 75 most abundant genera in the source filter materials (Bottom, Top_1 and Top2), and in each column after the 30 day experiment. Detailed operational conditions for each column are described in Table 1.

**Figure 3:**
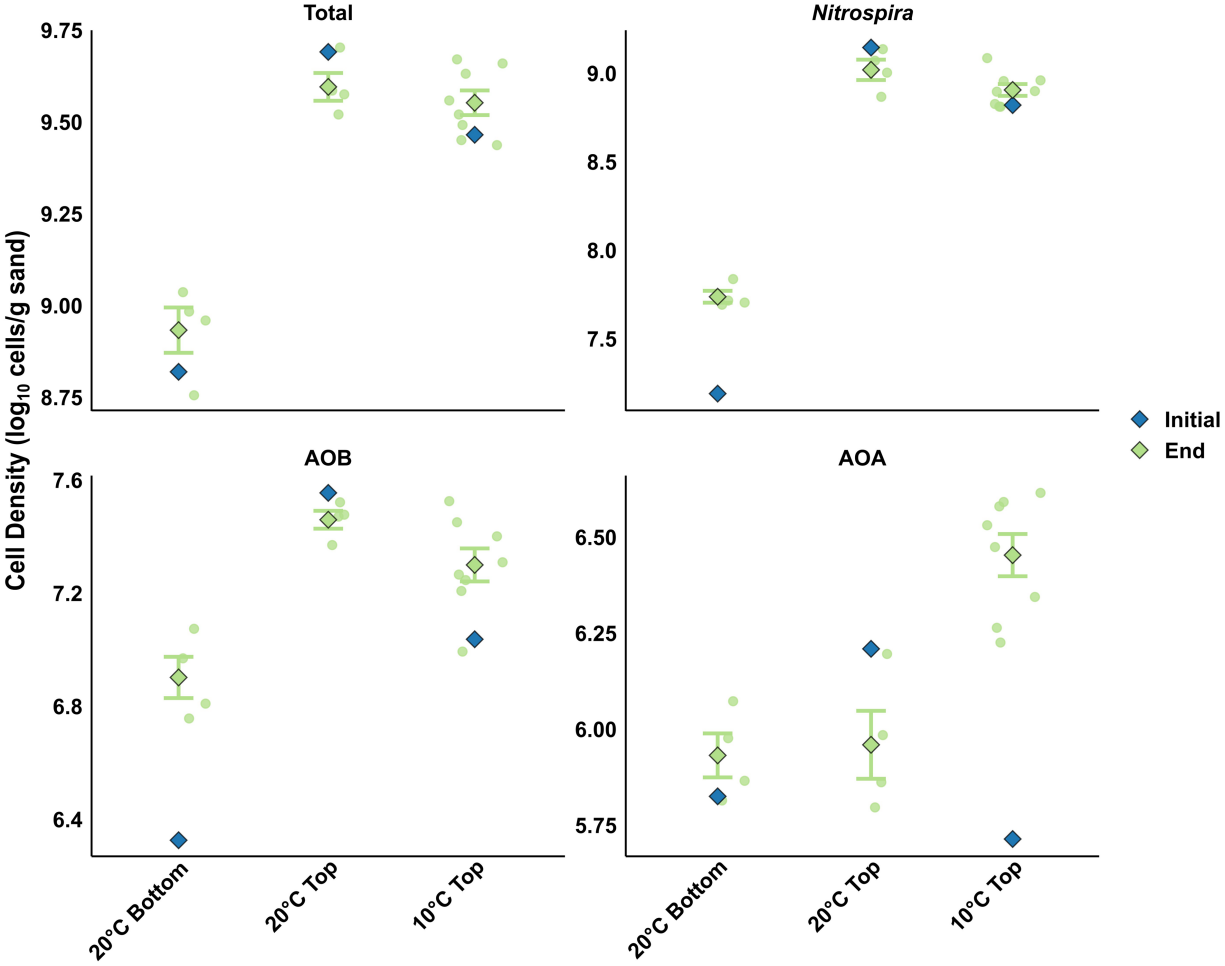
Cell density (log_10_ cells/g sand) determined by qPCR at the initial (blue color) and end (green color) of the experiment for the total community and the different nitrifier guild members under different temperature and filter layer. Data are grouped by filter layer origin and incubation temperature, reflecting the primary significant drivers of absolute abundance identified through linear modeling (Supplementary Table S4). Note the different scale on the *y*-axis.

The top and bottom sections of the full-scale RSF exhibited pronounced differences in community composition and microbial abundance prior to incubation (Figure 2A). Amplicon sequencing showed that the top-layer community was dominated by Proteobacteria (41%), Nitrospirae (29%), and Acidobacteria (13%). In contrast, the bottom layer community contained a higher proportion of Acidobacteria (22%) and a markedly lower abundance of Nitrospirae (4%) (Figure 2A). These differences were particularly evident among nitrifiers: *Nitrospira* spp. represented 28.8 ± 1.2% of the initial top-layer community but only 3.9% in the bottom layer (Figure 2B). *Nitrosomonas* spp. (AOB) were likewise in higher abundance in the top layer (0.9 ± 0.6%) relative to the bottom (0.1%), while ammonia-oxidizing archaea (AOA) constituted only a minor and similar fraction (0.03–0.1%) in both layers. Quantitative PCR confirmed that these compositional contrasts were mirrored by differences in absolute cell abundance. The top-layer material contained sixfold more total cells/g than the bottom layer (Figure 3). Among nitrifiers, *Nitrospira* abundance was 66-fold higher and AOB 11-fold higher in the top vs. bottom layer, whereas AOA differed only modestly (1.6-fold; Figure 3). These data establish that both the taxonomic composition and the biomass of nitrifying populations were stratified in the source filter material, forming the foundation for subsequent treatment-dependent dynamics described below.

### Distinct nitrifier guilds exhibit divergent responses to experimental disturbances

Linear modeling of final cell densities showed that filter layer origin remained the dominant factor controlling total microbial abundance (*p* < 0.001), with top-layer columns maintaining significantly higher densities (average of 3.6 × 10^9^ cells/g) than bottom-layer columns (7.9 × 10^8^ cells/g) throughout the experiment (Figure 3). Temperature (*p* = 0.41), oxygen availability (*p* = 0.49), water source (*p* = 0.19), and loading rate (*p* = 0.68) had no significant effect on total microbial density (Supplementary Table S4). Fold-change analysis revealed distinct growth dynamics between layers. Bottom-layer columns at 20 °C showed a 49% increase in total cell density, coinciding with the progressive improvement in ammonium removal (Figure 3). In contrast, top-layer columns at 20 °C exhibited a slight decline, while those at 10 °C showed modest growth (Figure 3). Among nitrifiers, *Nitrospira* spp. were the most strongly influenced by layer origin (p < 0.001). Bottom-layer columns showed marked *Nitrospira* proliferation, with (3.4–4.4)-fold increases in cell density (Log₂FC = 1.7–2.1; *p* < 0.01; Figure 3). In contrast, top-layer columns at 20 °C exhibited slight declines, whereas those at 10 °C remained stable (Figure 3). Despite the substantial *Nitrospira* growth in bottom layers, cell densities at the end of experiments in top-layer columns (average of 7.9 × 10^8^ cells/g) were still 19-fold higher than those in bottom-layer columns (4.2 × 10^7^ cells/g). Water source showed a marginal effect on *Nitrospira* abundance (*p* = 0.13), with after-filter effluent-fed columns maintaining slightly higher densities compared to influent (pre-filter effluent) or acetate-amended after-filter effluent. Neither temperature (*p* = 0.76), oxygen availability (*p* = 0.67), nor loading rate (*p* = 0.32) significantly affected *Nitrospira* densities (Supplementary Table S4). Patterns of AOB abundance closely mirrored those of *Nitrospira*, with layer origin as the dominant factor (p < 0.001). Top-layer columns sustained 3.6-fold higher AOB densities (2.1 × 10^7^ cells/g) than bottom-layer columns (6.0 × 10^6^ cells/g; Figure 3). Similar to *Nitrospira*, bottom-layer communities showed substantial AOB proliferation, with (3.4–5.6)-fold increases (Log₂FC = 1.8–2.5; *p* = 0.017). Likewise, top-layer columns at 20 °C exhibited slight AOB declines, whereas those at 10 °C showed modest increases (Figure 3). Neither temperature, oxygen availability, water source, or loading rate significantly affected AOB densities (all *p* > 0.17) (Supplementary Table S4).

In contrast to bacterial nitrifier behavior, AOA exhibited a fundamentally different set of responses. Temperature was the dominant factor influencing AOA abundance (*p* = 0.002), with 10 °C columns supporting an average 3.5-fold higher cell densities (3.0 × 10^6^ cells/g) than 20 °C columns (8.3 × 10^5^ cells/g; Figure 3). Layer origin, which dominated bacterial nitrifier distributions, had no significant effect on AOA abundance (*p* = 0.70), but these nitrifiers responded significantly to oxygen availability (*p* < 0.05), with 1.7-fold higher abundances under oxygen-limited conditions. Additionally, water source also influenced AOA populations (p < 0.05), with acetate-amended after-filter effluent supporting slightly lower densities compared to unspiked after-filter effluent or after-filter influent water. Loading rate showed no significant effect on AOA abundance (*p* = 0.37) (Supplementary Table S4). Collectively, these data indicate concurrent growth of all three nitrifier guild members under most experimental conditions, with no evidence of short-term competitive exclusion. Bacterial nitrifiers were primarily structured by filter layer origin and varied little across temperatures, while AOA exhibited a layer-independent, temperature-driven dynamics and were favored under oxygen-limited conditions.

### Enhanced ammonium removal is controlled by load-dependent biomass and activity dynamics

To determine whether enhanced ammonium removal was driven by increased nitrifier biomass or elevated cellular activity, we calculated cell-specific ammonium oxidation rates (SAOR). Initially, bottom-layer nitrifiers exhibited an order of magnitude higher per-cell activity than their top-layer counterparts across all loading conditions (Supplementary Figure S5). Despite this intrinsic physiological advantage, the substantially greater nitrifier biomass in the top layer drove superior bulk ammonium removal. Over the 30-day incubation, the response of SAOR was highly dependent on the ammonium loading. For bottom-layer communities, high loading triggered a significant physiological upregulation, elevating SAOR from 2.84 ± 0.34 to 4.27 ± 0.56 fmol NH₄^+^-N/cell/h. In contrast, at reference loading, SAOR remained statistically stable (from 1.10 ± 0.19 to 1.04 ± 0.14 fmol NH₄^+^-N/cell/h), indicating that improved ammonium removal rates (Supplementary Figure S3) were primarily driven by the observed nitrifier proliferation (Figure 3). For top-layer communities, where nitrifying biomass did not significantly change over the incubation period (Figure 3), the observed increase in per-cell activity (Supplementary Figure S5) appears to be the primary factor responsible for the improved ammonium removal rates (Supplementary Figure S3).

### Community restructuring and clade-specific dynamics reveal temperature-driven niche partitioning

**Figure 4:**
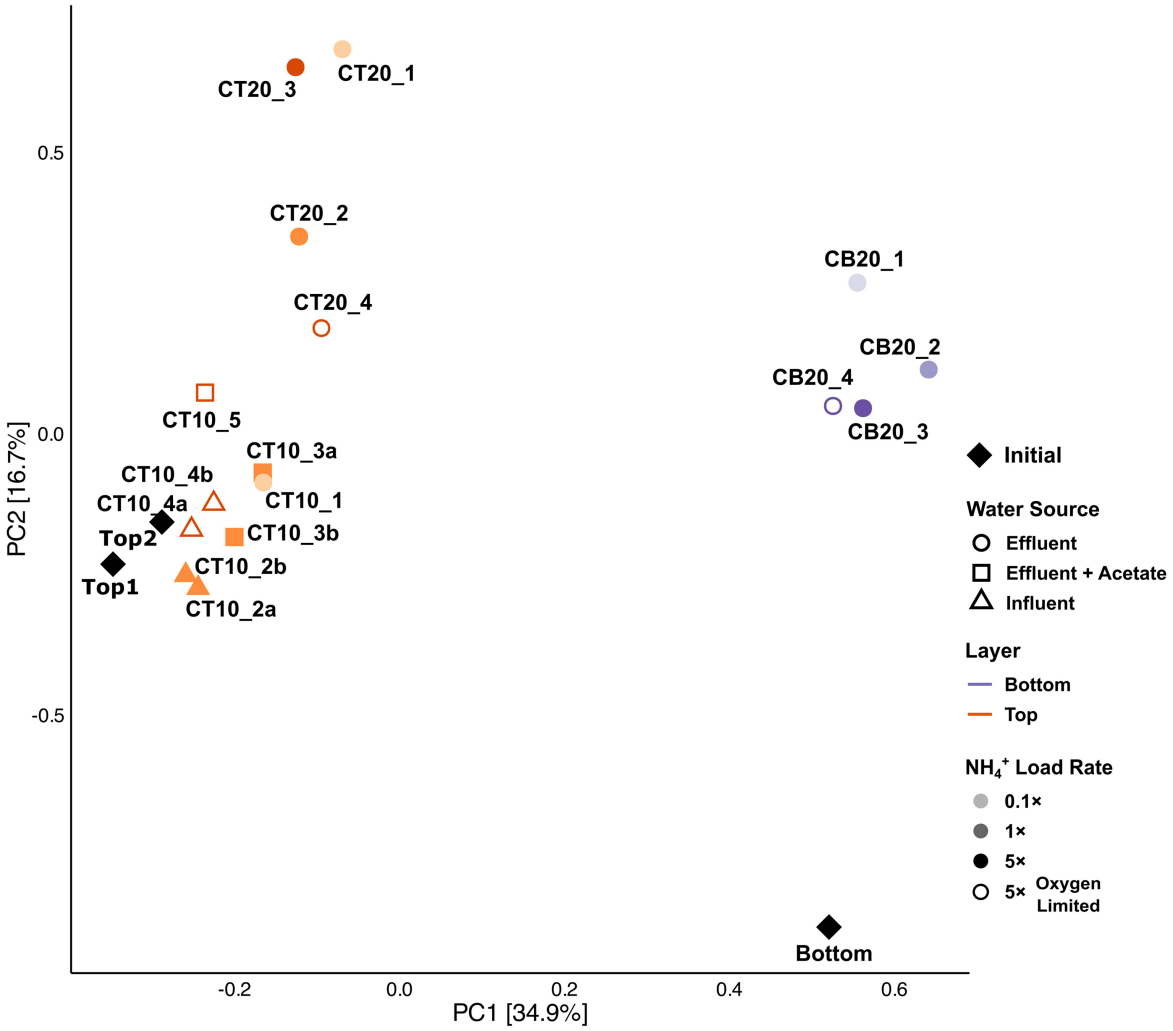
Principal component analysis based on the microbial community composition of the source filter materials and the filter materials at the end of the 30 day lab-scale experiments.

**Figure 5:**
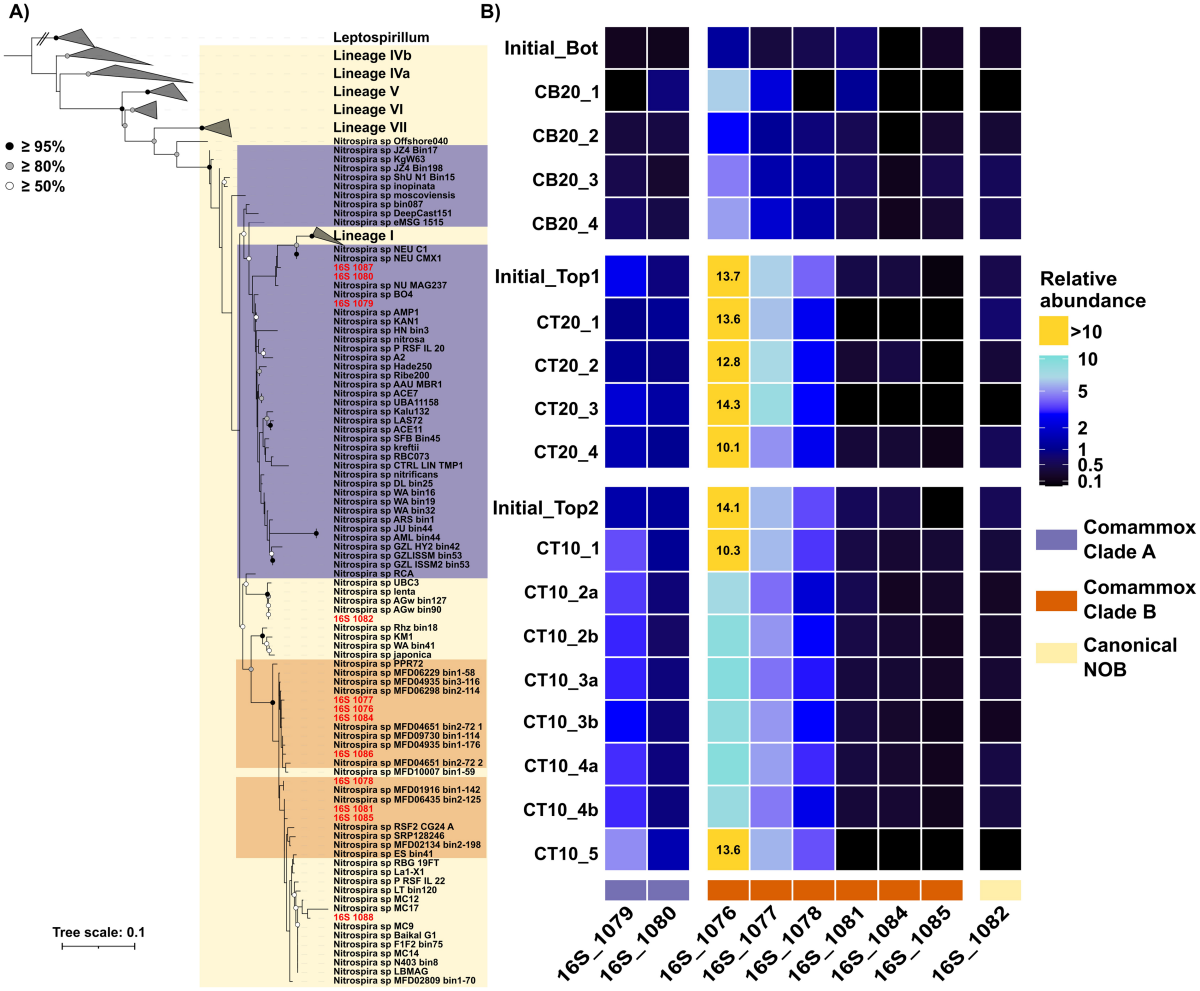
**(A)** Maximum-likelihood phylogenetic tree based on 16S rRNA gene sequences of *Nitrospira* (*Leptospirillum* as an outgroup). *Nitrospira* amplicon sequence variants recovered in this study with an average abundance > 0.01% and present in at least 10% of the samples are highlighted in red. *Nitrospira* lineages, and comammox clades assignment was conducted based on the affiliation on the genomes from where the 16S rRNA was recovered (further details in method section). **(B)** Heatmap showing the relative abundance of the dominant *Nitrospira* ASVs.

RSF community composition changed substantially over the 30-day period based on 16S rRNA gene amplicon sequencing, with the magnitude of restructuring mainly driven by temperature and layer origin (Figure 4). Columns operated at 20 °C experienced more pronounced compositional shifts from the initial communities than those at 10 °C, and bottom-layer columns underwent the most dramatic community reorganization (Figure 4). Sequencing results supported the qPCR-based quantitative trends and enabled resolution of clade-specific dynamics within *Nitrospira*, the dominant nitrifiers in all columns (Figure 5). The relative abundance of *Nitrospira* spp. increased significantly in bottom-layer columns (from 3.9% to 9.8 ± 2.2%), consistent with the substantial absolute growth detected by qPCR, while they remained similar in top-layer columns (from 28.8 ± 1.2% in the initial filter material to 26.0 ± 3.6% at the end of the experiment; Figure 2B). Comammox *Nitrospira* dominated the *Nitrospira* population according to 16S rRNA sequencing (Figure 5), consistent with previous metagenomic (92 ± 2% in top, and 95 ± 1% in bottom) and qPCR (70–99%) findings from the source filter (Fowler et al., 2018). Within this group, clade B was predominant (~85%), with clade A comprising ~15%, also in line with prior metagenomics and *amoA* amplicon data (Fowler et al., 2018). Clade-level dynamics, however, differed markedly between temperature treatments and filter-layer origin (Figure 5). In bottom-layer columns, differential abundance analysis identified two comammox clade B sequence variants (ASVs 16S_1076 and 16S_1077) that increased significantly during the incubation (padj < 0.05; Figure 5). In contrast, in top-layer columns we observed the opposite trend. At 10 °C, a consistent compositional adjustment occurred, characterized by a significant decline in the dominant clade B variant (16S_1076; from 14.1% to 9.6 ± 1.8%, padj < 0.0001) and a concurrent 2.4-fold enrichment of a clade A variant (16S_1079; from 1.4% to 3.4 ± 0.6%, padj < 0.0001), irrespective of ammonium loading, oxygen regime, or water source (Figure 5). Conversely, in top-layer columns operated at 20 °C, the same clade A variant slightly declined in relative abundance (from 2.5% to 1.4 ± 0.5%; Figure 5). No major compositional changes were detected among canonical *Nitrospira* ASVs.

Beyond nitrifiers, several taxa exhibited notable enrichment patterns. *Hydrogenophaga*, *Sphingobium*, and *Ferribacterium* showed increased relative abundances across all columns to varying degrees (Figure 2B). These genera, undetected in source filter material, showed substantial increases in columns operated at higher temperature, reaching highest relative abundances in the bottom-layer columns (*Hydrogenophaga*: 9.9 ± 4.5%; *Sphingobium*: 7.2 ± 2.5%; *Ferribacterium*: 4.5 ± 1.7%; Figure 2B). Other taxa exhibited condition-specific enrichment patterns. For instance, acetate amendment selectively stimulated *Acinetobacter* and *Pseudomonas* spp. in columns operated at reference loading rates (Figure 2B), while high ammonium loading conditions enriched *Novosphingobium* spp. (Figure 2B).

Together, these patterns reveal temperature- and substrate loading-dependent niche partitioning within comammox *Nitrospira* and accompanying shifts among heterotrophs, highlighting the rapid adaptive restructuring of RSF microbial communities under short-term environmental perturbations.

## Discussion

Lab-scale column experiments simulating groundwater-fed water production treatment demonstrate that rapid sand filter (RSF) microbial communities maintain robust nitrification capacity and functional stability across substantial short-term operational disturbances. Ammonium removal rates scaled proportionally with substrate loading under oxic conditions. In columns packed with top-layer material, removal rates reached levels five times higher than typical full-scale loading, consistent with previous investigations (Lee et al., 2014; Tatari et al., 2016). This linearity highlights a high physiological flexibility among the dominant nitrifiers. Our estimation of cell-specific rates suggests that these communities can efficiently exploit increased substrate fluxes by rapidly upregulating their metabolic activity; in the top-layer material, this mechanism supported higher bulk performance even in the absence of significant biomass growth. However, ammonium removal was ultimately constrained by oxygen availability, as removal rates plateaued once stoichiometric demand exceeded the bulk supply. This behavior is characteristic of co-diffusion biofilm systems (e.g., MBBRs) where nitrification becomes oxygen-limited once the bulk oxygen-to-ammonium ratio falls below stoichiometric requirements (Hem et al., 1994). Notably, neither temperature nor water matrix variations (e.g., acetate amendment) significantly affected ammonium removal, suggesting that RSF nitrification functions within a broad operational optimum maintained by oligotrophic, cold-adapted microbial consortia. The absence of a measurable temperature effect was surprising because enzyme kinetics of ammonia oxidation typically accelerate with increasing temperature (Huang et al., 2011; Razavi et al., 2015). However, this apparent insensitivity is consistent with the strong acclimation of the nitrifying communities to the *in situ* temperature regime of the full-scale filter (9–11 °C). Likewise, (Horak et al., 2013) showed that temperature did not have a significant effect on ammonia oxidation rates for incubation temperatures ranging from 8 to 20 °C in a natural marine community dominated by AOA from waters at ~8.5 °C. Similarly, acetate amendment had no significant effect on ammonium removal performance. Although elevated C/N ratios often impair nitrification by increasing competition for oxygen and surface attachment sites (Zhu and Chen, 2001; Hu et al., 2009), the modest acetate dose applied here (~1.5 mg/L) imposed only a minor additional oxygen demand (~1.2 mg/L O₂ per 1.5 mg acetate, compared with ~4.6 mg/L O₂ per 1 mg NH₄^+^-N oxidized). Effluent oxygen concentrations (4.5–5 mg/L) remained well above the half-saturation threshold for ammonia oxidation, confirming that nitrification was not oxygen-limited under these conditions. The enrichment of *Acinetobacter* (~2%) and *Pseudomonas* (~4.5%) in acetate-amended columns is consistent with the ability of these genera to utilize acetate (Salcedo-Vite et al., 2019; Dolan et al., 2020). Although effluent acetate concentrations were not directly monitored, the specific proliferation of these taxa suggests active consumption of the added carbon. This heterotrophic proliferation did not coincide with a decline in nitrifier abundances over the experimental period, suggesting that the carbon-fed guilds occupied ecological niches complementary to the nitrifying guilds. Interestingly, even under the most oxygen-demanding condition (i.e., columns fed with RSF effluent supplemented with 5 mg/L NH₄^+^ and 1.5 mg/L acetate) where stoichiometric oxygen demand (0.94 mg O₂/h) exceeded supply (0.42 mg O₂/h) by 124% and effluent oxygen concentrations dropped to near-anoxic levels (0.6–0.7 mg/L), ammonium removal efficiency and nitrifier abundance remained comparable to less oxygen-stressed treatments with the same loading but different water qualities. This resilience under severe oxygen limitation is consistent with two non-exclusive explanations. First, nitrifiers may have outcompeted aerobic heterotrophs for the limiting oxygen, since acetate-utilizing taxa such as *Acinetobacter* and *Pseudomonas*, abundant in non-oxygen-limited acetate treatments, were not detected under this oxygen-limited condition. Second, acetate may have been oxidized via alternative anaerobic respiratory pathways.

Although our analysis is limited to a two-layer comparison, the observed depth-dependent differences are consistent with previously reported vertical stratification of nitrifier guild density and compositions in full-scale and pilot-scale rapid sand filters (Lee et al., 2014; Gülay et al., 2016). This spatial partitioning is driven by steep physicochemical gradients created by sequential depletion of electron donors and acceptors with filter depth (Corbera-Rubio et al., 2023). Our results quantify this structure, showing that the top filter layer initially harbors sixfold higher total biomass and 66-fold higher abundance of *Nitrospira* than the bottom layer. This historical contingency determined the initial functional capacity, with top-layer columns consistently maintaining high ammonium removal performance while bottom-layer columns exhibited a distinct lag. However, this legacy effect was not deterministic, as both layers demonstrated significant adaptive potential through distinct mechanisms. In bottom-layer communities, adaptation was driven by a combination of biomass proliferation and physiological upregulation. While proliferation of *Nitrospira* and AOB occurred across all treatments, a clear physiological upregulation was specifically triggered under high loading rates, whereas specific activity remained stable under reference loading. In contrast, because nitrifying biomass in the top-layer material remained stable throughout the experiment, its ammonium removal improvements were primarily driven by increased per-cell activity. Interestingly, bottom-layer nitrifiers always displayed substantially higher cell-specific ammonium oxidation rates than top-layer nitrifiers. This likely reflects the massive standing stock of *Nitrospira* in the top layer (>10^9^ cells/g), which provides a large reserve capacity where the majority of cells may remain in a low-activity state while still ensuring robust bulk ammonium removal. Despite this higher per-cell efficiency and significant adaptive growth, bottom nitrifier guilds did not fully reach the bulk removal efficiencies of top nitrifier guilds because the absolute biomass difference remained too vast. This indicates a path-dependent assembly (priority effect) where the initial biomass distribution constrains system-level trajectories. Longer-term experiments would be needed to elucidate if a prolonged incubation time would allow the bottom community to eventually overcome this biomass gap and achieve complete ammonium removal.

Another important finding of this work is the decoupling of ecological drivers governing bacterial and archaeal nitrifiers co-existing in the RSF system. The abundance of bacterial nitrifiers (AOB and comammox *Nitrospira*) was overwhelmingly determined by their “legacy,” (i.e., their layer of origin), and appeared largely insensitive to temperature, oxygen availability. In contrast, AOA abundance was independent of layer origin and was instead strongly governed by environmental conditions. AOA responded significantly to temperature, oxygen and water source, apparently favored colder (10 °C) and oxygen-limited conditions. Despite these distinct drivers, we observed simultaneous growth and coexistence of all three nitrifier guild members (comammox, AOB, and AOA) across multiple conditions. This pattern is consistent with observations from full-scale RSFs, where these guilds frequently co-occur without evident exclusion (Palomo et al., 2022a), and contrasts with classical competition models predicting substrate-driven exclusion (Smith and Waltman, 1995; Rapaport and Veruete, 2019). According to kinetic theory, AOA, with very high ammonia affinities (*Kₘ*(app) ≈ 0.001–0.01 μM for groups I-II), should dominate at low free ammonia (FA), whereas AOB, with higher maximum growth rates but lower affinities (*Kₘ*(app) ≈ 0.5–30 μM), should prevail at elevated FA levels, and comammox occupy an intermediate niche (*Kₘ*(app) ≈ 0.04–0.06 μM) (Jung et al., 2022). Our experiments spanned FA concentrations from 0.0001 μM (top-layer, 10 °C, 1 × loading) to 2.77 μM (bottom-layer, 20 °C, 5 × loading). Although comammox *Nitrospira* remained the dominant nitrifier under all conditions, AOA exhibited their highest fold-increase at the lowest FA treatments, and AOB showed the greatest proliferation in high-FA columns, consistent with their predicted kinetic preferences but without evidence of guild replacement. This suggests that substrate availability rather than competition shaped growth responses. Several factors likely contribute to this stable coexistence. First, kinetic parameters are derived from a few cultivated representatives and may not reflect the physiological diversity within each guild. For instance, only a few comammox species have been physiologically characterized (Kits et al., 2017; Sakoula et al., 2021). In addition, the dominant AOB populations in this system are phylogenetically affiliated with *Nitrosomonas* cluster 6a and *Nitrosomonas* sp. PY1, AOB with relatively high substrate affinity (*Km*(app) = 0.5–5 μM) (Jung et al., 2022; Kikuchi et al., 2023), which could blur expected competitive boundaries. Second, spatial heterogeneity within biofilms creates steep O₂ and NH₃ gradients that can enable fine-scale niche partitioning not captured by bulk measurements. Third, the 30-day timescale may be insufficient for competitive exclusion given slow nitrifier growth rates (doubling times estimated in columns are 10–21 days) and the strong initial dominance of *Nitrospira*. Together, these mechanisms could explain why guild-specific kinetic predictions alone cannot capture observed community dynamics. In structured biofilms, spatial microheterogeneity, physiological trade-offs, and historical contingency may act jointly to sustain a resilient and functionally redundant nitrifier assemblage across a broad environmental spectrum.

Beyond intra-guild coexistence, shifts within comammox *Nitrospira* exhibited clade-specific dynamics. At 10 °C, a comammox clade A variant increased consistently (~2.4-fold) while the previously dominant clade B sequence declined, regardless of water source or loading regime. This shift was not observed at 20 °C, despite 10 °C reflecting the *in situ* RSF temperature. This turnover cannot be readily attributed to temperature adaptation alone, since clade B dominates in the full-scale filter operating under similar thermal conditions. Alternatively, stochastic processes or top-down biotic interactions, such as phage predation, which can selectively impact *Nitrospira* populations in engineered systems (Palomo et al., 2023), or competition with co-occurring heterotrophs (Zhu and Chen, 2001), could have selectively constrained the clade B dominant variant in the columns. Future work should track clade-resolved growth rates and gene expression during controlled transitions from field to lab conditions across temperatures to disentangle whether this represents a transient stress response or a fundamental difference in ecological fitness.

While this study establishes that niche complementarity underpins the functional stability of RSF nitrification, the specific ecological rules driving selection and assembly within the ammonia-oxidizing guild require further exploration. To move beyond broad guild-level insights, future research must address two critical questions regarding community trajectories and niche definitions. First, it remains to be determined whether RSF community trajectories are deterministic or historically contingent. Our 30-day timeframe captured rapid adaptation but could not resolve whether the bottom-layer community would eventually converge to the high-efficiency state of the top layer. Long-term incubations (>6 months) are necessary to test whether initial biomass distributions create permanent alternative stable states (priority effects) or if sufficient time allows for functional convergence regardless of legacy. Second, to properly assign realized niches to specific guild members, we must distinguish active contributors from the dormant reserve fraction. Since DNA-based abundance does not equate to physiological activity, as evidenced by the high-density but low-activity top-layer populations, future work should integrate metatranscriptomics (Lawson et al., 2017) or stable-isotope probing (SIP) (Gülay et al., 2019). This would allow for the unequivocal linking of function to specific clades under in situ conditions, validating the activity-based niche partitioning proposed here. Finally, coupling these approaches with genome-scale metabolic modeling (Basile et al., 2020) could elucidate the cryptic biotic interactions, such as cross-feeding or competition, that ultimately shape the coexistence of these functionally redundant guilds.

Overall, this study provides a high-resolution model of the RSF nitrification engine, bridging guild member-specific ecophysiology with community-level disturbance response. We demonstrate that the functional stability of these engineered ecosystems is underpinned by a sophisticated biological insurance policy: functional redundancy is maintained through niche partitioning at multiple taxonomic levels. Though all members of the nitrifier guild coexisted across different conditions without evident competitive exclusion, substrate-dependent niche partitioning was observed with free-ammonia concentrations spanning three orders of magnitude. In addition, our findings have direct engineering and ecological implications. For operators, our results indicate that nitrification performance in RSFs is relatively robust to short-term variations in temperature and influent water chemistry, with no measurable decline across the tested conditions. In contrast, stoichiometric oxygen availability provides an absolute limit on ammonium removal, consistent with previous observations in biological filtration (Steele et al., 2006; Lytle et al., 2013). Furthermore, the strong differences in nitrifiers density between filter layers at different depths have further implications for maintenance: backwashing or media replacement that disrupts the biologically active top layer can temporarily impair function. In contrast, strategies preserving top layer microbiomes (e.g., shallow backwash, top layer reseeding) should help maintain consistent performance. Ecologically, we show that bacterial (AOB, *Nitrospira*) and archaeal (AOA) nitrifiers are governed by fundamentally different assembly rules in these systems. The coexistence of multiple nitrifier taxa and clades under controlled laboratory conditions confirms patterns observed in full-scale RSFs, and validates functional redundancy as a fundamental mechanism ensuring stable ecosystem services in engineered water treatment systems facing climate variability.

## Data Availability

The sequencing data generated in this study have been deposited in the NCBI Genebank under BioProject accession PRJNA1357839.

## Footnotes

1
https://github.com/ardagulay/CaRcone---Community-average-rRNA-gene-copy-nr-estimator
