# Supplementary material for: Functional redundancy and niche complementarity maintain nitrification stability in rapid sand filters
Source: Front Microbiol. 2026 Jan 21;16:1741059. doi: 10.3389/fmicb.2025.1741059 (PMC12868214; doi:10.3389/fmicb.2025.1741059)
Supplement: Supplementary file 1 [file Data_Sheet_1.PDF]

Functional Redundancy and Niche Complementarity Maintain Nitrification Stability in Rapid Sand Filters

Alejandro Palomo[1,2,3], S Jane Fowler[4,3], Ibrahim M. Nemer[3], Borja Valverde-Pérez[3], Yan Zheng[1,2], Yunjie Ma[1,2], Barth F Smets[5,3]

[1] State Key Laboratory of Soil Pollution Control and Safety, Southern University of Science and Technology, Shenzhen 518055, China

[2] Guangdong Provincial Key Laboratory of Soil and Groundwater Pollution Control, School of Environmental Science and Engineering, Southern University of Science and Technology, Shenzhen 518055, China

[3] Department of Environmental and Resource Engineering, Technical University of Denmark, Bygningstorvet 115, 2800 Kgs. Lyngby, Denmark

[4] Department of Biological Sciences, Simon Fraser University, Canada

[5] Department of Biological and Chemical Engineering, Environmental Engineering, Aarhus University, Nordre Ringgade 1, Aarhus, 8000, Denmark

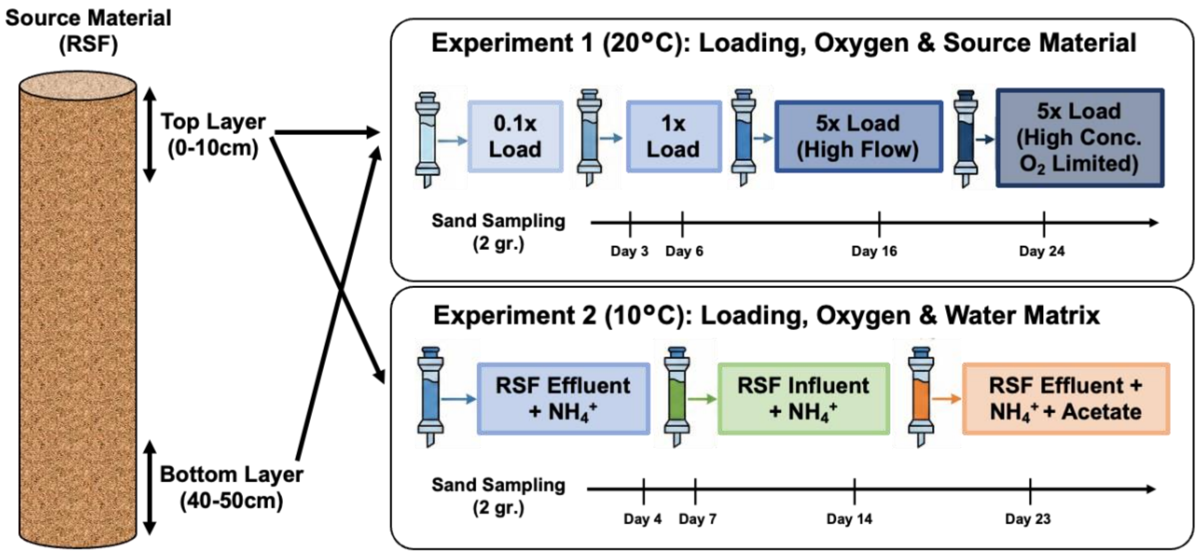

Supplementary Figure S1. Summary of the experimental design.

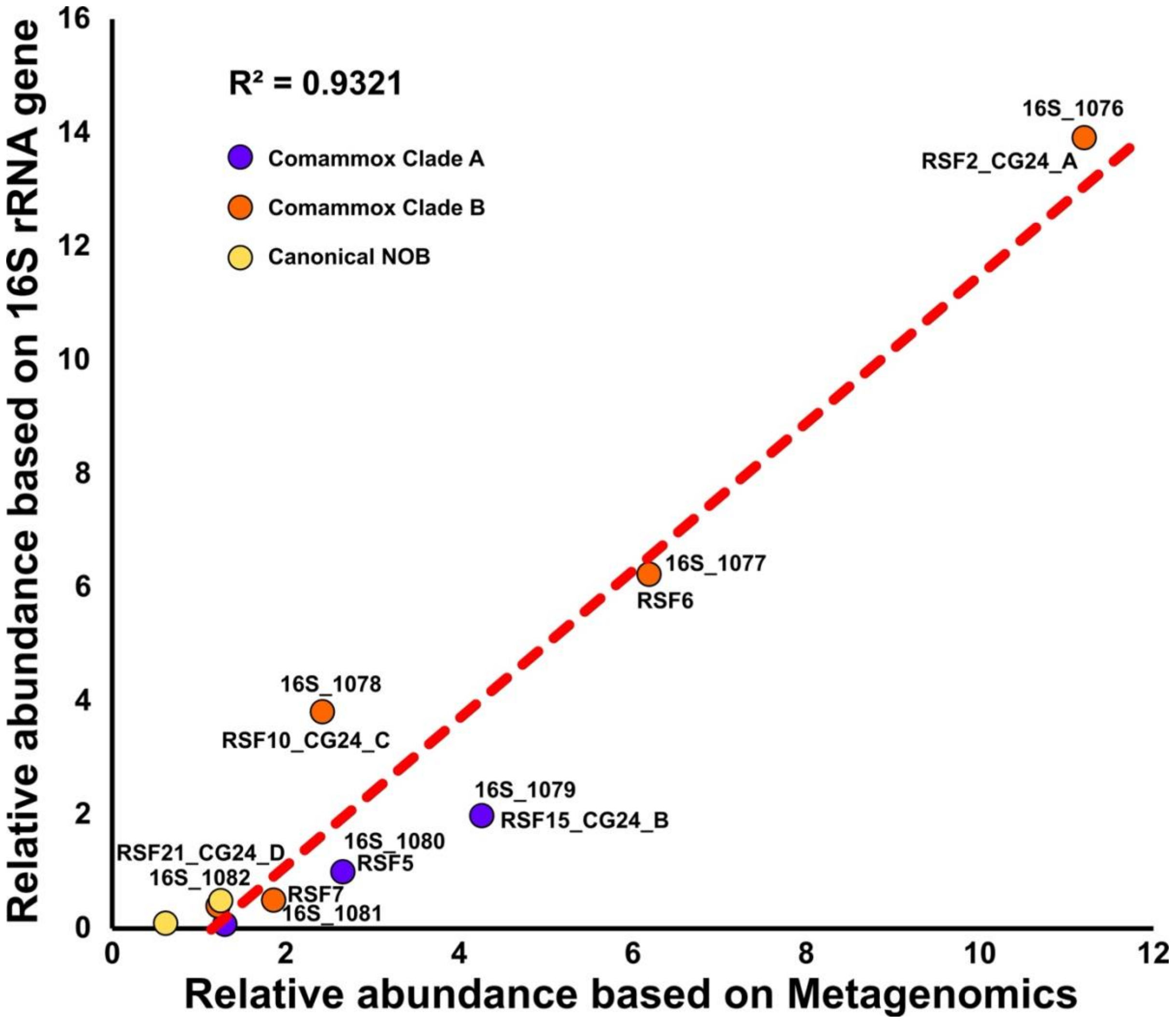

Supplementary Figure S2. Relative abundance (%) of *Nitrospira* in the upper 10 cm of a rapid-sand filter (Islevbro waterworks). Coloured dots represent metagenome-assembled genomes (MAGs) and amplicon sequence variants (ASVs). MAG abundances were calculated from read mapping (n = 3 metagenomes); ASV abundances are based on 16S rRNA gene amplicons (n = 2 amplicon libraries). Purple, comammox clade A; orange, comammox clade B; yellow, canonical NOB.

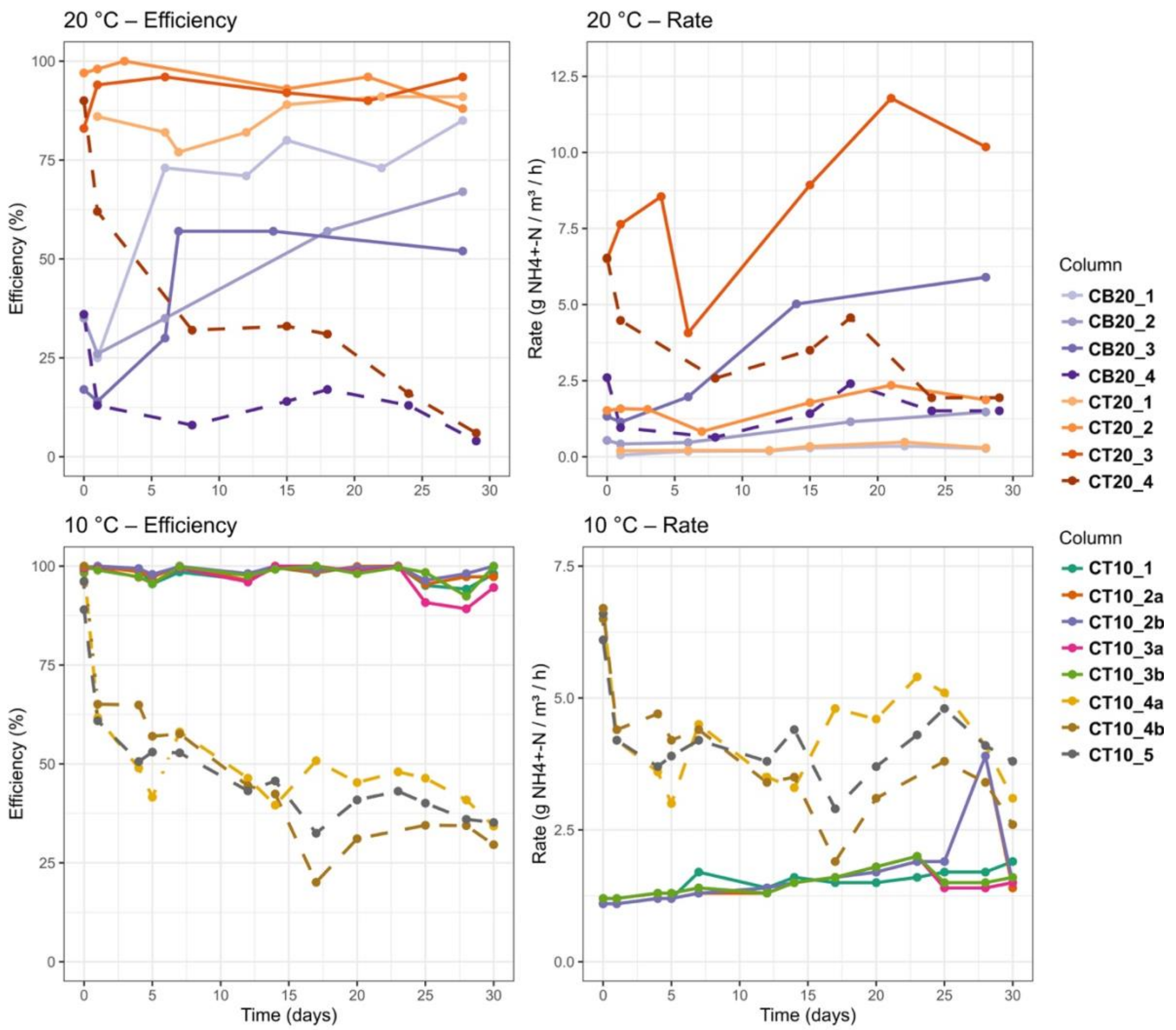

Supplementary Figure S3. Ammonium removal efficiency and removal rate over time in the different columns. Detailed operational conditions for each column are described in Table 1.

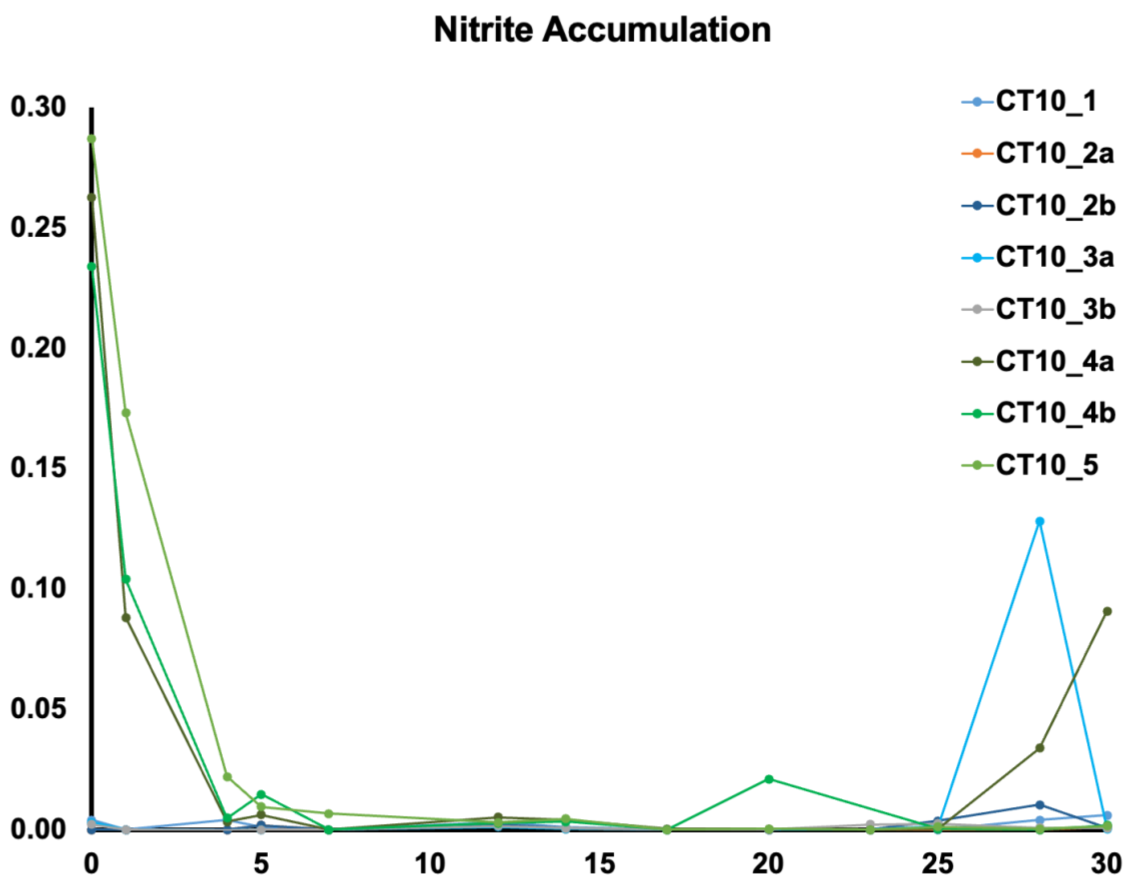

Supplementary Figure S4. Nitrite accumulation (mg/L) over time (days) in the effluent of the different columns. Detailed operational conditions for each column are described in Table 1.

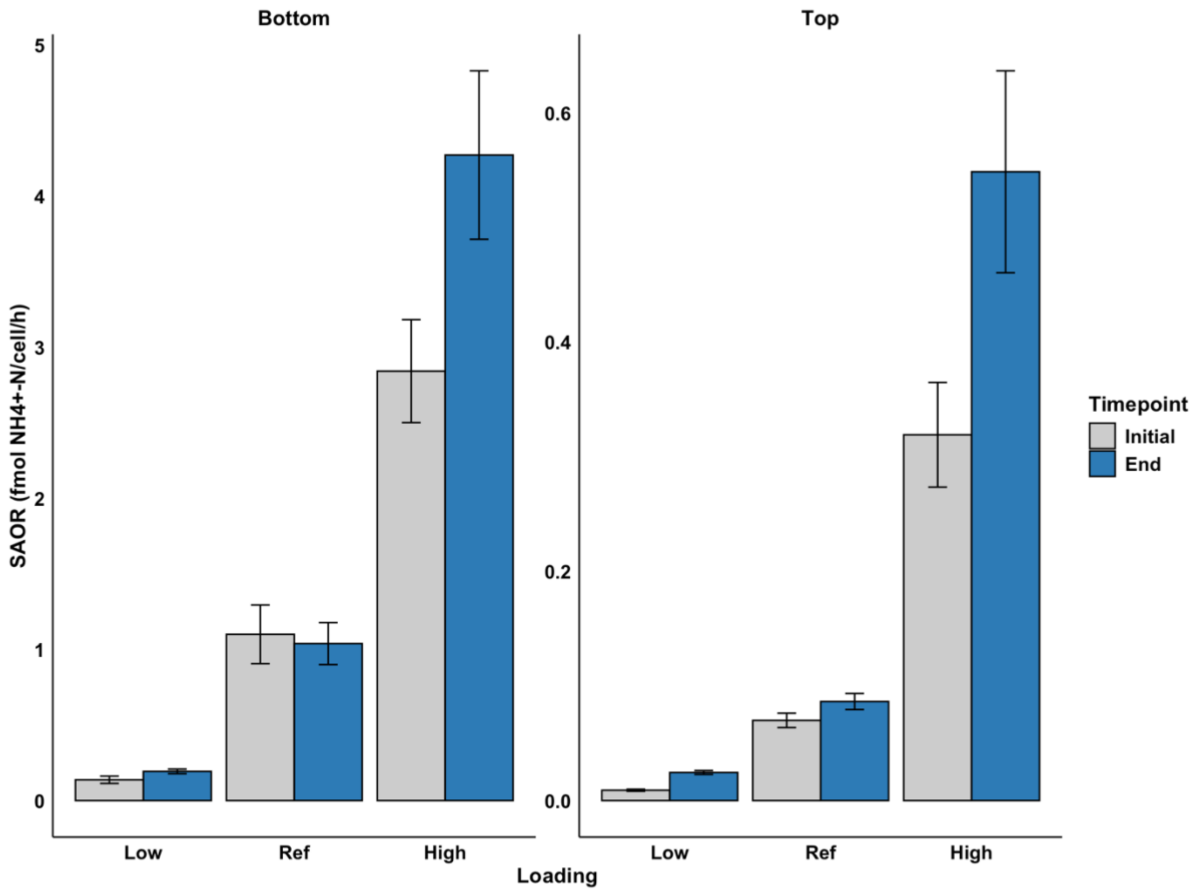

Supplementary Figure S5. Apparent cell-specific ammonium removal rates (SAOR) in rapid sand filter columns inoculated with top and bottom filter material under low, reference, and high ammonium loading regimes. Rates are shown for the initial phase (based on inoculum biomass) and at the end of the 30-day experiment. Note the different scale on the y-axis for Bottom and Top columns.

Supplementary Table S1. Primers and thermocycling protocols used in this study

**Gene target**	**Primer name**	**Sequence**	**Ta (°C)**	**Reference**
**16S Bacteria[1] (Sequencing)**	Bakt 341F<br>Bakt 805R	CCTAYGGGRBGCASCAG<br>GGACTACNNGGGTATCTAAT	56	(Yu et al., 2005)
**16S Bacteria[2] (qPCR)**	1055F<br>1392R	ATGGCTGTCGTCAGCT<br>ACGGGCGGTGTGTAC	55	(Ferris and Muyzer, 1996)<br>(Lane, 1991)
**16S AOB[2]**	CTO189FA/B<br>CTO189FC<br>RT1R	GGAGRAAAGCAGGGGATCG<br>GGAGGAAAGTAGGGGATCG<br>CGTCCTCTCAGACCARCTACTG	60	(Kowalchuk et al., 1997)<br>(Hermansson and Lindgren, 2001)
**16S *Nitrospira*[2]**	Nspra675F<br>Nspra746R	GCGGTGAAATGCGTAGAKATCG<br>TCAGCGTCAGRWAYGTTCCAGAG	64	(Graham et al., 2007)
**amoA AOA**	CrenamoA23F<br>CrenamoA616R	ATGGTCTGGCTWAGACG<br>GCCATCCATCTGTATGTCCA	55	(Tourna et al., 2008)

[1] For thermocycling protocol see Gulay et al. 2016

[2]Thermocycling protocol: 94°C 5:00, 40 cycles of 94°C 30s, Ta 30s, 72°C 1:00, melt curve (70°C -95°C) 0.2°C/s gradient.

Supplementary Table S2. Genome identity of the 16S rRNA genes with highest similarity to the *Nitrospira* ASVs recovered in this study. Colors denote genome affiliation: comammox clade A (purple), clade B (orange) and canonical NOB (yellow).

	1st Hit (BLAST)			2nd Hit (BLAST)		
ASV ID	Similarity	Accession NCBI	Genome name	Similarity	Accession NCBI	Genome name
16S_1076	100%	JACMLT010000000	Nitrospira sp. ES-bin-41	100%	CAVZXH010000000	Nitrospira sp. MFD04651.bin.2.72
16S_1077	100%	JACMLT010000000	Nitrospira sp. ES-bin-41	99.77%	CAVZXH010000000	Nitrospira sp. MFD04651.bin.2.72
16S_1078	100%	CAWGEO010000000	Nitrospira sp. MFD01916.bin.1.142	100%	CAMBDY010000000	Nitrospira_sp_LT_bin120
16S_1079	100%	JABFRI010000000	Nitrospira sp. P-RSF-IL-20	100%	JAFEBH010000000	Nitrospira sp. KAN1
16S_1080	99.77%	JABFRI010000000	Nitrospira sp. P-RSF-IL-20	99.77%	JAFEBH010000000	Nitrospira sp. KAN1
16S_1081	100%	CAVUMA010000000	Nitrospira sp. MFD06435.bin.2.125	99.77%	JBBRRI010000000	Nitrospira sp. LA1-X1
16S_1082	100%	OUNR01000000	Nitrospira lenta			
16S_1084	99.77%	JACMLT010000000	Nitrospira sp. ES-bin-41	99.77%	CAVZXH010000000	Nitrospira sp. MFD04651.bin.2.72
16S_1085	99.77%	CAVUMA010000000	Nitrospira sp. MFD06435.bin.2.125	99.54%	JBBRRI010000000	Nitrospira sp. LA1-X1
16S_1086	99.77%	CAWBQY010000000	Nitrospira sp. MFD09730.bin.1.114	99.54%	JACMLT010000000	Nitrospira sp. ES-bin-41
16S_1087	99.54%	CZPZ01000000	Ca. Nitrospira nitrificans			
16S_1088	99.77%	JBJXEO010000000	Nitrospira sp. F1F2_bin75	99.77%	JAWSTL010000000	Nitrospira sp. N403_bin8

Supplementary Table S3. Relative abundance (%) of *Nitrospira*, comammox *Nitrospira*, and comammox clades A and B in top and bottom layers of a RSF at Islevbro waterworks. Data compiled from the present study and earlier investigations in the same filter.

	Top				Bottom				
	Nitrospira **(% total community)**	**Comammox (% *Nitrospira*)**	**Clade B (% Comammox)**	**Clade A (% Comammox)**	***Nitrospira*** **(% total community)**	**Comammox (% *Nitrospira*)**	**Clade B (% Comammox)**	**Clade A (% Comammox)**	**Reference**
**16S rRNA gene sequencing**	28.8 ± 1.2	97.8 ± 0.1	89.2 ± 2.0	10.8 ± 2.0	3.9	86.7	91.8	8.2	This study
**qPCR**	25.6 ± 4.1	-	-	-	2.3	-	-	-	This study
**Metagenomics**	36.7 ± 2.6	92.5 ± 2.2	71.8 ± 6.2	28.2 ± 6.2	11.1 ± 6.8	95.4 ± 0.3	72.9 ± 0.4	27.1 ± 0.4	Palomo et al., 2016; Palomo et al., 2022^
**AmoA-targeted qPCR and sequencing**	33 - 43%	> 90%	75 - 80%	20 - 25%	-	-	-	-	Fowler et al., 2017

^NCBI Project: PRJNA384587; samples can be found in SRA: SRR5739198, SRR5739199, SRR5739200, SRR5739201, SRR5739202, SRR5739203

Supplementary Table S4. Summary of Type III ANOVA for linear models of final microbial cell densities.

Target Group	Factor	F-value	p-value	Significance
Total Community	Layer (Origin)	102.99	7.60E-06	***
	Loading Rate	0.40	0.68	ns
	Oxygen	0.52	0.49	ns
	Temperature	0.75	0.41	ns
	Water Source	2.06	0.19	ns
Nitrospira	Layer (Origin)	468.57	2.19E-08	***
	Loading Rate	1.31	0.32	ns
	Oxygen	0.19	0.67	ns
	Temperature	0.10	0.76	ns
	Water Source	2.64	0.13	ns
AOB	Layer (Origin)	34.10	3.88E-04	***
	Loading Rate	1.44	0.29	ns
	Oxygen	1.60	0.24	ns
	Temperature	0.03	0.86	ns
	Water Source	1.81	0.22	ns
AOA	Layer (Origin)	0.15	0.70	ns
	Loading Rate	1.12	0.37	ns
	Oxygen	6.59	0.03	*
	Temperature	20.83	1.84E-03	**
	Water Source	7.27	0.02	*

'***' < 0.001; '**' <0.01; '*' < 0.05; 'ns' >0.05
